# Rheological Properties of DNA Molecules in Solution: Molecular Weight and Entanglement Influences

**DOI:** 10.3390/polym8080279

**Published:** 2016-08-03

**Authors:** Lourdes Mónica Bravo-Anaya, Frédéric Pignon, Félix Armando Soltero Martínez, Marguerite Rinaudo

**Affiliations:** 1 Laboratoire Rhéologie et Procédés (LRP), University Grenoble Alpes, Grenoble F-38000, France; monik_ayanami@hotmail.com (L.M.B.-A.); frederic.pignon@univ-grenoble-alpes.fr (F.P.); 2 Centre National de la Recherche Scientifique (CNRS), Laboratoire Rhéologie et Procédés (LRP), Grenoble F-38000, France; 3 Departamento de Ingeniería Química, Universidad de Guadalajara, Blvd. M. García Barragán, Guadalajara C.P. 44430, Mexico; jfasm@hotmail.com; 4 Biomaterials applications, 6 rue Lesdiguières, Grenoble F-38000, France

**Keywords:** low and high molecular weight DNA, hydrodynamic behavior, overlap parameter *C*_DNA_[η]

## Abstract

Molecular weight, stiffness, temperature, and polymer and ionic concentrations are known to widely influence the viscosity of polymer solutions. Additionally, polymer molecular weight—which is related to its dimensions in solution—is one of its most important characteristics. In this communication, low molecular weight DNA from salmon sperm was purified and then studied in solutions in a wide concentration range (between 0.5 and 1600 mg/mL). The intrinsic viscosity of this low molecular weight DNA sample was firstly determined and the evidence of the overlap concentration was detected around the concentration of 125 mg/mL. The chain characteristics of these short molecules were studied in terms of the influence of their molecular weight on the solution viscosities and on the overlap parameter *C*_DNA_[η]. Furthermore, to complete previously reported experimental data, solutions of a large molecular weight DNA from calf-thymus were studied in a high concentration range (up to 40 mg/mL). The rheological behavior is discussed in terms of the generalized master curve obtained from the variation of the specific viscosity at zero shear rate (η_sp,0_) as a function of *C*_DNA_[η].

## 1. Introduction

Up to now, two of the most-studied fundamental properties of biomacromolecules such as DNA, proteins, and polysaccharides have been the hydrodynamic and conformational properties [1,2]. For these purposes, the intrinsic viscosities and the radii of gyration have been the most often determined [1,3,4]. In the field of biotechnology, changes in the hydrodynamic properties of these kinds of biomolecules are useful in the development and study of targeting pharmaceutical molecules [5,6]. Moreover, small fragments of DNA, oligonucleotides, or short biomolecules are important in nanotechnology studies, due to their contribution to several biological processes such as DNA packing around histones, compaction, gene transcription, gene delivery via small volume carriers for gene therapy, among others [7,8,9,10].

Recently, Pan et al. [11] reported that zero shear rate viscosity of semi-dilute unentangled DNA solutions have a power law dependence on the scaled concentration C/C* (where *C* is the polymer mass concentration and C* is the overlap concentration), with an effective exponent depending on the solvent quality parameter *z*. In their work, they determined the θ*-*temperature of dilute DNA solutions in the presence of salt excess, but they never considered the stiffness of the DNA molecule. Recently, a detailed study of the rheological behavior of high molecular weight DNA (calf-thymus) solutions was reported, as well as evidence of the two critical concentrations of the system—i.e., the overlap and the entanglement concentrations (C* and C**, respectively) [1]. Furthermore, a generalized master curve was proposed from the variation of the specific viscosity at zero shear rate (η_sp,0_) as a function of the overlap parameter (*C*[η]) in a range of *C*_DNA_[η] values up to 40.

In this paper, a novel representation of the η_sp,0_ vs. *C*[η] relationship based on the expression previously reported by Kwei et al. [12] is proposed, taking into account the rheological behavior of the semi-dilute regime with entanglements—i.e., for concentrations over C** and *C*_DNA_[η] values up to 160. Additionally, chain characteristics of very short molecules (only some tens of base pairs), which are of great importance to molecular or cell biology, [13,14,15] are studied. The η_sp,0_ vs. *C*[η] relationship was then found to be applicable to DNA chains with a molecular weight lower than the one corresponding to the persistence length of 50 nm, where DNA molecules behave as semi-flexible rods.

## 2. Materials and Methods

### 2.1. Materials and Solutions Preparation

Low molecular weight (LMW) DNA from salmon sperm, purchased as lyophilized powder, was purified and then turned into its sodium salt form. The purification process is described in the following section. Anhydrous NaCl was used to prepare a solvent solution at a concentration of 0.1 M. A series of low molecular weight DNA solutions was prepared in the concentration range from 0.5 to 1600 mg/mL. High molecular weight (HMW, *M*_w_ = 6,559,500 g/mol [1]) DNA samples from calf thymus DNA were used to prepare solutions in a concentration range from 0.01 to 40 mg/mL. A buffer solution prepared with Tris-HCl (100 mM) and EDTA (10 mM) was used to maintain a pH of 7.3. These HMW DNA solutions were prepared with a solvent consisting of a 9:1 ratio of HPLC water and the Tris–HCl/EDTA buffer (TE buffer). All reagents were provided by Sigma-Aldrich Company (Toluca, México). All solutions were prepared with HPLC-grade water. To prevent water evaporation, the vials were closed and sealed with Parafilm^®^ (Bemis NA, Neenah, WI, USA). All solutions were stored at a temperature of 4 °C in order to prevent DNA degradation and were left for a period of at least one week for stabilization and homogenization.

### 2.2. Purification of Low Molecular DNA from Salmon Sperm

Low molecular weight lyophilized DNA powder from salmon sperm was firstly dissolved in water, adding a stoichiometric amount of NaOH 1.0 N. A precipitation with ethanol (60% *v*/*v*) was then performed in the presence of salt excess (NaCl 1.0 M) to exchange the possible divalent counterions and to recover the sodium salt of DNA, which must be water soluble [16]. Finally, DNA was recovered by solvent exchange with ethanol–water mixtures up to 100% in ethanol, and drying at 40 °C.

### 2.3. Capillary Measurements

Viscosity measurements of low molecular weight DNA solutions with concentrations between 0.5 and 100 mg/mL were carried out with a capillary viscometer Micro-Ubbelohde (SCHOTT Instruments GmbH, Mainz, Germany) connected with a semi-automatic chronometer ViscoClock (SCHOTT Instruments GmbH, Mainz, Germany) at a temperature of 20 °C. The selected capillary (No. 501 01) has a diameter of 0.53 ± 0.01 mm and a constant *K* equal to 0.005.

### 2.4. Rheological Measurements

The rheological behavior of low and high molecular weight DNA solutions was studied through flow measurements by using a DHR-3 rheometer from the TA Instruments Company (New Castle, DE, USA). Three different geometries were used, depending on DNA molecular weight and on the solution concentration: (1) a steel cone with a 60 mm diameter and an angle of 2° was used for low and high molecular weight DNA solutions with concentrations in the dilute regime and in the semi-dilute regime without entanglements (*C*_DNA_ < C**); (2) a steel cone with a 40 mm diameter and an angle of 2° was used for low molecular weight DNA solutions with concentrations higher than 1000 mg/mL and for high molecular weight DNA solutions with concentrations between 2 mg/mL and 10 mg/mL; and (3) a rough steel cone and plate for high molecular weight DNA solutions with concentrations higher than 10 mg/mL, the cone having a 35 mm diameter and an angle of 2°. Simple shear steady state measurements were performed in a shear rate range from 1 × 10^−3^ to 1000 s^−1^, using five points per decade. Each sweep was performed at a temperature of 20 ± 0.1 °C, controlled by a Peltier plate.

## 3. Results and Discussion

### 3.1. Low Molecular Weight DNA Sample Characterization 

After purification, the commercial low molecular weight DNA from salmon sperm was characterized through UV-Vis and ^1^H NMR measurements to confirm its structure [17,18]. When dissolved in water, the structure of this purified LMW DNA sample corresponds to that of an oligonucleotide in a semi-denatured conformation. The absorbance ratio A_260_/A_280_ was measured on a purified sample dissolved in NaCl 0.1 M, from which was possible to evaluate its purity (i.e., 1.70 ± 0.01), in good agreement with the literature [1,19].

Then, since the DNA molecule bears one formal negative charge per nucleotide, its conformation is certainly sensitive to changes in the ionic strength, in the number of base pairs, and in the molecular weight [20,21,22]. In this manner, the information about intrinsic viscosity is necessary to determine and to understand the hydrodynamic properties of the DNA molecule under specific conditions. Therefore, viscosity capillary measurements were performed in order to determine the intrinsic viscosity of the low molecular weight DNA sample in NaCl 0.1 M at 20 °C. Reduced viscosities were calculated according to Equation (1), and were plotted as a function of DNA concentration (*C*_DNA_) following the Huggins relation [1,23].

η_red_ = η_sp_/*C* = [η] + k’[η]^2^*C* (1)

where *C* is the polymer concentration (g/mL), η_red_ is the reduced viscosity, η_sp_ is the specific viscosity (corresponding to (η − η_s_)/η_s_, where η_s_ is the solvent viscosity), [η] is the intrinsic viscosity (mL/g) and k’ is the Huggins constant.

The intrinsic viscosity, (8.49 mL/g) was obtained from extrapolation to zero concentration. It is worth mentioning that low values of intrinsic viscosities between 7.5 mL/g and 160 mL/g were previously reported for oligonucleotides and oligosaccharides [2,24,25]. A recently established Mark-Houwink relation (Equation (2) for the low molecular weight DNA range—between 10 and 3000 bp (base pair)—was used to estimate the viscometric-average molecular weight of the sample [2].

[η] = 3.5 × 10^−4^*M*^1.05^ (2)

Thus, the calculated viscometric-average molecular weight for this sample is equal to 15,000 g/mol, equivalent to around 23 bp (estimated by taking the average weight of a DNA nucleotide in salt solution as 325 g/mol).

Finally, the overlap concentration (C*) was estimated through the deviation from linear behavior in the dilute regime (i.e., 125 mg/mL), which is also in good agreement with the value obtained from the relation to C*~[η]^−1^.

### 3.2. Rheological Behavior of Low Molecular Weight DNA

**Figure 1:**
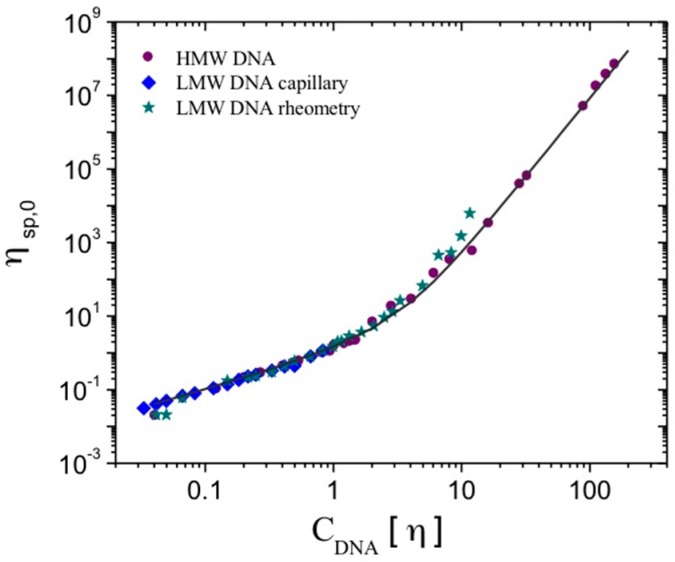
Dependence of the specific viscosity at zero shear rate (η_sp,o_) as a function of the overlap parameter *C*_DNA_[η] for low molecular weight (LMW) DNA from salmon sperm and high molecular weight (HMW) DNA from calf-thymus at different concentrations *C*_DNA_. The solid line represents the master curve expressed by Equation (3).

It is well known that long DNA chains like T2, T4, T5, and T7 viruses [26,27], and even fragments of whole chains viruses [28], present a viscoelastic behavior; however, as the chains get smaller, the critical value for the shear rate—characterizing the appearance of non-Newtonian behavior—increases. This transition from Newtonian to non-Newtonian behavior also depends on the polymer concentration. In this work, the viscosity of low molecular weight DNA solutions at low shear rates was studied through flow measurements as a function of DNA concentration. The behavior of the solution remains Newtonian in all the domains of the selected shear rates—i.e., up to 100 s^−1^. Then, the variation of the specific viscosity as a function of polymer concentration was analyzed in terms of the overlap parameter (*C*_DNA_[η]) by means of the generalized master curve, initially proposed for hyaluronans with various molecular weights. The importance of this approach is recognized since the intrinsic viscosity values ([η]) also contain information regarding the stiffness of the polymer [29,30]. All the data obtained for the specific viscosity determined by capillary and rheological measurements for low molecular weight DNA samples and for the specific viscosity determined in the Newtonian plateau for high molecular DNA samples are plotted together and collapse in the same curve (Figure 1).

It should be pointed out that knowing the intrinsic viscosity of the polymer, it becomes possible to calculate the specific viscosity at zero shear rate (η_sp,0_) for a given polymer concentration and to estimate the critical concentrations of the system—i.e., the overlap concentration (C*~[η]^−1^) and the entanglement concentration (C**, corresponding to *C*[η]~10).

Equation (3) presents a novel representation of the relationship between the specific viscosity and the overlap parameter, which is based on the expression previously proposed by Kwei et al. for hyaluronan samples [12].

η_sp_ = *C*[η] [1 + *k*_1_(*C*[η]) + *k*_2_(*C*[η])^2^ + *k*_3_(*C*[η])^3.3^] (3)

where *k*_1_ represents the Huggins constant, which for several water soluble polymers is equal to 0.4, *k*_2_ = (*k*_1_)^2^/2! = 0.08 and *k*_3_ = 0.0213.

In this equation, the fourth term represents the behavior of the semi-dilute entangled regime, with a final slope equal to 4.3 at high polymer concentrations (around *C*[η] > 10), instead of the value proposed by Kwei et al. [12] with *k*_3_ = (*k*_1_)^2^/3!. This slope value is in good agreement with the power law established for the regime over the entanglement concentration C**, for which η/η_Rouse_ varies as (*C*/C**)^3.4^ and *η* varies as (*C*/*Ce*)^4.42^ [31,32]. Furthermore, the reported behavior of the experimental values for the specific viscosities of hyaluronan and xanthan samples at high polymer concentrations in 0.1 N NaCl followed the slopes of 4.18 and 4.24, respectively [16,33].

### 3.3. Rheological Behavior of High Molecular Weight DNA in the High Concentration Domain

In order to prevent wall-slip phenomena while performing steady state flow measurements of high molecular weight DNA in the concentration domain larger than 10 mg/mL [34], a cone–plate geometry with roughened base plate and cone was selected. The roughening of the geometry surfaces has been satisfactorily applied by several authors and with different materials, like sandblasting or sandpaper [35,36,37].

At DNA concentrations higher than 10 mg/mL, there is a large non-Newtonian domain, implying the use of a model to describe the rheological curve. Then, the viscosity dependence on shear rate for several DNA solutions with concentrations between 15 and 40 mg/mL was analyzed with the Cross model, given by Equation (4) [38] (the goodness of the fits having an average *R*^2^ of 0.98 ± 0.01).

(4) $$\frac{\eta -\eta_{\infty }}{\eta_{0}-\eta_{\infty }}=\frac{1}{1+{\left(K\overset{•}{\gamma }\right)}^{m}}$$

where $\overset{•}{\gamma }$ is the shear rate, η_0_ is the zero shear-rate viscosity, η_∞_ is the viscosity at infinite shear-rate, *m* is a dimensionless parameter related to the degree of shear thinning, and *K* has the dimensions of time.

At larger polymer concentrations, the specific viscosity from the zero shear-rate viscosity determined through the Cross model follows the previously-presented master curve up to *C*_DNA_[η] = 160 (Figure 1). The slope value for specific viscosity as a function of *C*_DNA_[η] for these highly concentrated calf-thymus DNA solutions corresponds to that of the semi-dilute regime with entanglements, and remains 4.3.

## 4. Conclusions

A commercial low molecular weight DNA sample from salmon sperm was purified, characterized, and then studied in solution in a wide concentration range between 0.5 and 1600 mg/mL. The intrinsic viscosity was found to be equal to 8.49 mL/g, which is in the range of the values of oligonucleotides and oligosaccharides. The viscometric-average molecular weight value for this sample corresponds to 15,000 g/mol. The evidence of the overlap concentration was detected around the concentration of 125 mg/mL. The behavior of these low molecular weight DNA solutions remains Newtonian in all domains of the selected shear rates (i.e., up to 100 s^−1^).

All of the data for the specific viscosity at zero shear rate obtained for this LMW DNA were plotted together as a function of the overlap parameter, and collapse on the curve obtained for the HMW DNA. The proposed relationship between η_sp,o_ and *C*_DNA_[η] is then valid for DNA chains with molecular weights lower than 1 × 10^5^ g/mol, where chains are in an almost rod-like state, and for DNA chains having higher molecular weights, where molecules behave as worm-like chains.

For high molecular weight DNA solutions from calf-thymus with concentrations up to *C*_DNA_[η] = 160, the zero shear-rate viscosity determined through the Cross model follows the improved master curve. Up to *C*[η] = 160, the slope value remains at 4.3, with a behavior corresponding to that of the semi-dilute regime with entanglements.

## Abbreviations

The following abbreviations are used in this manuscript:

DNA: Deoxyribonucleic Acid
LMW: Low Molecular Weight
HMW: High Molecular Weight
TE: TrisHCl-EDTA
bp: Base pair
